# Antifungal Secondary Metabolites Produced by the Fungal Endophytes: Chemical Diversity and Potential Use in the Development of Biopesticides

**DOI:** 10.3389/fmicb.2021.689527

**Published:** 2021-06-21

**Authors:** Kuo Xu, Xiu-Qi Li, Dong-Lin Zhao, Peng Zhang

**Affiliations:** Tobacco Research Institute of Chinese Academy of Agricultural Sciences, Qingdao, China

**Keywords:** fungal endophytes, secondary metabolites, chemical diversity, phytopathogenic fungi, antifungal activities, biopesticides

## Abstract

Plant diseases caused by phytopathogenic fungi can lead to huge losses in the agricultural fields and therefore remain a continuous threat to the global food security. Chemical-based fungicides contributed significantly in securing crop production. However, indiscriminate application of fungicides has led to increased chemical resistance and potential risks to human health and environment. Thus, there is an urgent need for searching for new bioactive natural products and developing them into new biopesticides. Fungal endophytes, microorganisms that reside in the fresh tissues of living plants, are regarded as untapped sources of novel natural products for exploitation in agriculture and/or medicine. Chemical examination of endophytic fungi has yielded enormous antifungal natural products with potential use in the development of biopesticides. This review summarizes a total of 132 antifungal metabolites isolated from fungal endophytes in the past two decades. The emphasis is on the unique chemical diversity of these metabolic products, together with their relevant antifungal properties. Moreover, some “star molecules,” such as griseofulvin and trichothecene, as well as their synthetic derivatives that possess high potential as candidates of new natural fungicides, are also presented herein.

## Introduction

Plant diseases caused by phytopathogenic fungi are continuing to be a huge threat in the agricultural fields. It is estimated that the global loss caused by plant diseases is more than 20% of the crop yield in the major food and cash crops worldwide (Kim et al., 2004). Fungal pathogens were responsible for the considerable postharvest losses of grain crops, fruits, and vegetables, which, in addition to causing decay, can produce mycotoxins that are harmful to humans and animals (Bai et al., 2013; Shi et al., 2017). Therefore, effective and sustained control of these fungal pathogens has become an urgent task. Chemical control has been widely adopted in crop production. During the past years, a great variety of chemical fungicides (agrochemicals), such as thiophanate-methyl, carbendazim, and imazalil, are designed and applied to control these diseases. It is indisputable that these chemicals have resulted in substantial increases in productivity and contributed significantly in agricultural industry. However, these most-applied chemical fungicides have been limited by many serious problems. Indiscriminate use of these fungicides has led to the appearance of pathogens with multiple fungicide resistances, which further complicated the management of the diseases (Talibi et al., 2014). Furthermore, repeated and exclusive use of fungicides is increasingly restricted owing to their undesirable effects on non-target organisms (carcinogenicity, high and acute residual toxicity) and potential risks to environmental pollution (long degradation period) (Bai et al., 2013; Talibi et al., 2014). Thus, these synthetic chemical fungicides are subject to registration and permission for use, and are even no longer authorized in various countries.

Based on above questions, the challenge is to develop new and eco-friendly alternatives for the safe control of these pathogens, which pose low risk to human health and environment. Nowadays, many promising biological approaches were considered to be potential alternatives to synthetic fungicides, including (i) use of biocontrol microorganisms, (ii) application of naturally sourced metabolites, and (iii) induction of natural resistance (Talibi et al., 2014). Among them, naturally sourced secondary metabolites from microbes have attracted great attention. Microorganisms are well known for their ability to synthesize bioactive secondary metabolites, which have provided abundant chemical entities for pharmaceuticals and agrochemicals. Many natural antifungal fungicides, such as kasugamycin, polyoxins, validamycin, and blasticidin-S, have been obtained from microbial resources (Copping and Duke, 2007; Wang et al., 2016).

Fungal endophytes, microorganisms that asymptomatically reside in the internal tissues of plants, have been widely distributed in almost every plant and are rich in diversity (Jia et al., 2016). Endophytic fungi have a close and complex interaction with their hosts, which involve mutualism, antagonism and rarely parasitism (Gouda et al., 2016). These species are known to promote host growth and gain essential nutrition. They also provide tolerance to plants against various types of abiotic and/or biotic stresses. Most importantly, they possess the ability to produce plenty of structurally diverse and biologically active secondary metabolites to protect their hosts from pathogenic microorganisms and pests. In this sense, endophytic fungi are a treasure source of searching for novel secondary metabolites with immense potential agricultural applications. It has been reported that a large number of metabolites with different chemical skeletons have been deciphered from endophytic fungi, such as alkaloids, terpenoids, steroids, peptides, benzopyranones, quinones, and isocoumarins (Gouda et al., 2016). Chemically speaking, the discovery of these metabolites provided impressive chemical basis in the development of agrochemicals. Moreover, most of them exhibited promising bioactivities, such as antifungal, antibacterial, herbicidal, nematocidal, insecticidal, and other agricultural activities.

Therefore, the topical subject of antifungal secondary metabolites produced by fungal endophytes has been searched and analyzed. The literature search was conducted using the combined keywords “antifungal,” “secondary metabolites,” and “endophytic fungus” in the databases such as Web of Science, Google Scholar, and SciFinder Scholar, with a previously reported search method (Zhang et al., 2020). As a result, a total of 132 metabolites with anti-phytopathogenic activities from fungal endophytes in the past two decades (covering from 2000 to 2020) were included in this review. The present compounds possess diverse chemical structures, which were classified into alkaloids (including cytochalasins, indoles, diketopiperazines, and other nitrogen-containing compounds), terpenoids, polyketides (quinones, macrolides, benzopyrones and unsaturated lactones), and other miscellaneous compounds within a biogenetic context. Moreover, we also describe the sources, the producing strains, target plant pathogenic fungi, and their potential use as lead compounds in the development of biopesticides.

## Confused Issues Need to Be Addressed

Prior to the beginning of this review, there are three issues that need to be addressed. (i) The level of antifungal potency should be standardized. As we can see from literatures, the literatures are replete with uncalibrated potency descriptors, including ‘mild, moderate, strong, pronounced, significant, remarkable, and potent.’ Apparently these descriptions confused the readers that which level means strong and which should be defined as weak. The unified standardization has not yet formed since the definition of the level of bioactivity just represents the individual judgment of the authors. However, in this review, the antifungal potency was distinguished as ‘potent,’ ‘moderate,’ and ‘weak’ based on the comparison with positive controls. We discretionarily define ‘potent’ as the antifungal activity higher than that of the positive controls, ‘moderate’ as the bioactivity equal to that of the positive controls, and ‘weak’ as that of lower than positive controls (or inactive) throughout this review. (ii) Quantitative comparisons of the results of the bioactivity tests between different studies are problematic. As the fungal pathogens used in bioassays may have different provenance and viability/sensitivity with different assay protocols, the comparisons should be qualified by consideration of positive/negative controls, and even then should be best limited within individual studies, rather than between different studies. (iii) The selection of different positive controls may lead to radically different results. Commercial fungicides were generally chosen as positive controls. However, since they belong to different chemical classes, they may present distinct potency and mechanisms of action.

## Chemical Diversity of Antifungal Secondary Metabolites From Endophytic Fungi

### Cytochalasin Alkaloids

**FIGURE 1:**
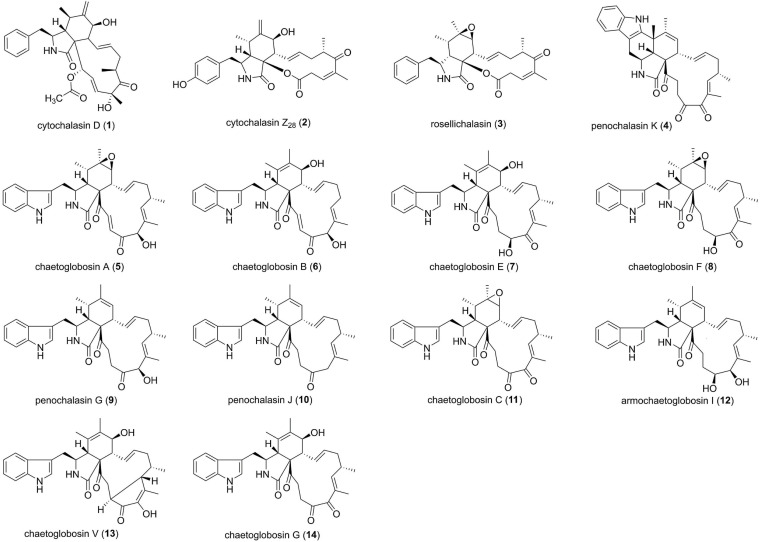
Cytochalasin alkaloids characterized from endophytic fungi (1–14).

The cytochalasin alkaloids are a class of structurally related fungal metabolic products. To date, more than 300 cytochalasin analogs have been isolated from many genera of ascomycetes and basidiomycetes, including *Aspergillus, Chaetomium, Penicillium, Phomopsis, Phoma, Spicaria, Xylaria*, and so on (Wei et al., 2017). Structurally, cytochalasins are characterized by a highly substituted perhydroisoindol-1-one moiety which is fused with a 9- to 15-membered macrocyclic ring. Many of cytochalasins exhibit a wide range of biological activities, such as cytotoxic, antimicrobial, and phytotoxic properties (Wei et al., 2017; Xu et al., 2017). 14 cytochalasins (Figure 1) isolated from fungal endophytes were reported to possess moderate to potent antifungal activity. Cytochalasin D (1) was produced by various endophytic fungi *Xylaria* sp., which were isolated from leaves of guarana plant (Elias et al., 2018). 1 showed fungistatic activity against the phytopathogen *Colletotrichum gloeosporioides*, which causes the anthracnose disease, with an MIC of 2.46 mM. Commercial fungicides captan and difenoconazole were applied as positive controls (MICs 16.63 and 0.02 mM, respectively) (Elias et al., 2018). Bioassay-guided separation of *Xylaria* sp. XC-16, an endophyte from *Toona sinensis* led to the discovery of five agriculturally active cytochalasin alkaloids, including a new compound cytochalasin Z_28_ (2) (Zhang Q. et al., 2014). Compound 2 showed potent fungicidal effect (MIC = 12.5 μM) against the phytopathogen *Gibberella saubinetti*, which was better than that of the positive control hymexazol (MIC = 25 μM) (Zhang Q. et al., 2014). Chemical investigation of the biocontrol potential endophytic fungus *Aspergillus capensis* CanS-34A in *Brassica napus* has resulted in the isolation and identification the antifungal metabolite rosellichalasin (3) (Qin et al., 2019). 3 inhibited the plant pathogenic fungi *Botrytis cinerea, Monilinia fructicola, Sclerotinia sclerotiorum*, and *S. trifoliorum* with the EC_50_ values of 36.8, 87.1, 5.3, and 41.1 μM, respectively. Thus, *S. sclerotiorum* was the most sensitive target fungus (Qin et al., 2019). A new chaetoglobosin, penochalasin K (4) possessing a rare six-cyclic 6/5/6/5/6/13 fused ring system, was isolated from the solid culture of the mangrove endophytic fungus *Penicillium chrysogenum* V11 (Zhu et al., 2017b). Compound 4 displayed potent selective activities against *C. gloeosporioides* and *Rhizoctonia solani*, with MIC values of 6.13 and 12.26 μM, respectively, which were about ten-fold and two-fold better than that of the positive control carbendazim (Zhu et al., 2017b). Five metabolites, chaetoglobosins A (5), B (6), E (7), F (8), and penochalasin G (9), were obtained from endophytic *Chaetomium globosum*, isolating from the seeds of *Panax notoginseng* (Li et al., 2016). Some of them exhibited remarkable inhibition against phytopathogenic fungi causing root rot disease. For example, chaetoglobosin E (7) and penochalasin G (9) indicated potent inhibition against *Epicoccum nigrum* with the MICs < 2 μM (Li et al., 2016). A new chaetoglobosin named penochalasin J (10), as well as two known chaetoglobosins, chaetoglobosin C (11) and armochaetoglobosin I (12), were isolated from the mangrove endophytic fungus *P. chrysogenum* V11 (Huang et al., 2016). Compound 10 showed more potent antifungal activity against plant pathogen *C. gloeosporioides* with an MIC value of 25.08 μM, than the positive control carbendazim (MIC = 65.38 μM). Simultaneously, compounds 11 and 12 remarkably inhibited *R. solani*, with MIC values of 23.66 and 12.11 μM, respectively (Huang et al., 2016). Chaetoglobosins V (13) and G (14) were isolated from the culture of the endophytic fungus *C. globosum*, associated with the leaves of *Ginkgo biloba* tree (Xue et al., 2012). Compounds 13 and 14 exhibited potent antifungal activity against *Alternaria solani*, with MICs of 47.3 μM, while 14 also possessed potent activity against *A. alternate*, with an MIC of 47.3 μM (Xue et al., 2012). In total, the above results indicated that cytochalasin alkaloids could be used as fungicides or as leads of new fungicides to the related phytopathogenic fungi.

### Alkaloids

**FIGURE 2:**
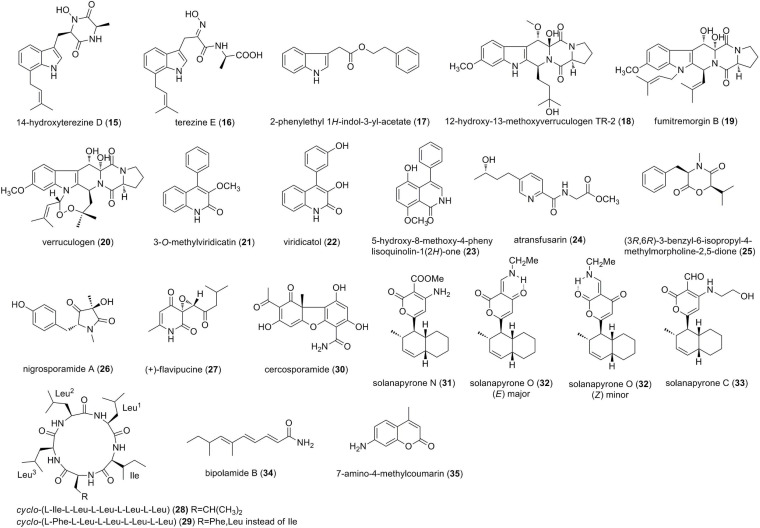
Other alkaloids characterized from endophytic fungi (15–35).

Except for the cytochalasins, alkaloids also include indoles, diketopiperazines, cyclopeptides, amides, and other N-containing compounds (Figure 2). Two prenylated tryptophan analogs, a previously reported 14-hydroxyterezine D (15) and a new terezine E (16), were isolated from an endophytic *Mucor* sp. from the medicinal plant *Centaurea stoebe* (Abdou et al., 2018). Both 15 and 16 exerted weak antifungal efficacy against *Aspergillus terreus*, with MICs of 127.8 and 111.2 μM, respectively (Abdou et al., 2018). Chemical investigation of the endophytic fungus *C. gloeosporioides* from the leaves of *Michelia champaca* resulted in the isolation of one new indole alkaloid, 2-phenylethyl 1*H*-indol-3-yl-acetate (17) (Chapla et al., 2014). 17 exhibited moderate activity against *Cladosporium cladosporioides* and *C. sphaerospermum* at 5 μg, which was comparable to that observed for the positive control nystatin (Chapla et al., 2014). Three prenylated indole diketopiperazine alkaloids, 12β-hydroxy-13α-methoxyverruculogen TR-2 (18), fumitremorgin B (19), and verruculogen (20), were isolated from *Aspergillus fumigatus* LN-4, an endophytic fungus isolated from the stem bark of *Melia azedarach* (Li et al., 2012). Compounds 18-20 showed broad-spectrum anti-phytopathogenic activities against eight fungi (*B. cinerea, A. solani, A. alternata, C. gloeosporioides, Fusarium solani, F. oxysporum* f. sp. *niveum, F. oxysporum* f. sp. *vasinfectum*, and *G. saubinettii*), with MIC values of 13.7-100 μM, which were comparable to the positive controls carbendazim and hymexazol (Li et al., 2012). Two quinolinones 3-O-methylviridicatin (21) and viridicatol (22), together with a new isoquinolone alkaloid named 5-hydroxy-8-methoxy-4-pheny lisoquinolin-1(2*H*)-one (23) were isolated from the fermentation of an endophytic fungus *Penicillium* sp. R22 in *Nerium indicum* (Ma et al., 2018). Compounds 21-23 exhibited weak to moderate antifungal activities against *Alternaria brassicae*, *A. alternate, B. cinerea*, and *Valsa mali* with MIC values of 124.3, 123.3, and 116.8 μM, respectively (Ma et al., 2018). A new fusaric acid derivative, atransfusarin (24), and (3*R*,6*R*)-3-benzyl-6-isopropyl-4-methylmorpholine-2,5-dione (25) were isolated from the culture of an endophyte *Alternaria atrans* MP-7, associated with the medicinal plant *Psidium guajava* (Yang Z. et al., 2019). Compound 25 exhibited potent antifungal activities against *A. solani, C. gloeosporioides*, and *Phyricularia grisea* with MICs of 6.25 μM, better than that of a broad-spectrum fungicide carbendazim. In contrast, 24 only exerted weak activities against *B. cinerea* and *A. solani* (MIC = 50 μM) (Yang Z. et al., 2019). A new pyrrolidinone derivative, named nigrosporamide A (26), was obtained from an endophytic fungus *Nigrospora sphaerica* ZMT05, which was isolated from *Oxya chinensis Thunberg* (Zhu et al., 2017a). 26 exhibited higher antifungal activity against *C. gloeosporioides* with an MIC value of 25.14 μM, than the positive control triadimefon (MIC = 272.39 μM) (Zhu et al., 2017a). It should be pointed out that the MIC of the positive control triadimefon (272.39 μM) seems to be outside the error measurements, probably because triadimefon is insensitive to *C. gloeosporioides*. As we discussed above, other appropriate positive controls should be rechose. From the culture extracts of the endophytic fungus *Phoma* sp. isolated from the plant *Salsola oppositifolia*, a new pyridione epoxide derivative, (+)-flavipucine (27), was isolated and characterized (Loesgen et al., 2011). This metabolite showed strong antifungal inhibition down to 7.81 ppm (Inhibitory concentration, 90% of growth inhibition) against *Phytophthora infestans* and down to 31.3 ppm against *Septoria tritici* (Loesgen et al., 2011). Two cyclic pentapeptides 28 and 29 were isolated from *Cryptosporiopsis* sp., an endophytic fungus from *Zanthoxylum leprieurii* (Rutaceae) (Talontsi et al., 2012). Compounds 28 and 29 exhibited motility inhibitory and lytic activities against *Plasmopara viticola* zoospores, a grapevine downy mildew pathogen, at 17.7-44.3 μM. Moreover, both of them also displayed potent inhibitory effects against mycelial growth of *Pythium ultimum, Aphanomyces cochlioides*, and *R. solani* (Talontsi et al., 2012). A metabolite cercosporamide (30) was isolated from the endophytic fungus *Cadophora orchidicola* from *Kalimeris indica* (Wang et al., 2019). Antifungal assay revealed that 30 had potent growth inhibition against five plant pathogens, *Pestalotia diospyri, B. cinerea, Fusarium oxysporum, Sclerotium rolfsii*, and *Penicillum digitatum*, with EC_50_ values of 16.0 × 10^–3^, 1.8, 2.8, 2.89, and 20.2 μM, respectively (Wang et al., 2019). Two new solanapyrone analogs, solanapyrones N (31) and O (32), and the known solanapyrone C (33), were isolated from *Nigrospora* sp. YB-141, an endophytic fungus obtained from *Azadirachta indica* (Wu et al., 2009). Compound 32 was regarded as an inseparable mixture of (*E*)-32 and (*Z*)-32. Compound 31 showed moderate activity against *Penicillium islandicum* at equivalent concentration of 98.6 μM compared with the positive control nystatin (Wu et al., 2009). Bipolamide B (34), a new triene fatty acid amide, was discovered from the endophytic fungus *Bipolaris* sp. MU34 of Thai medicinal plants *Gynura hispida* (Siriwach et al., 2014). Compound 34 showed weak to meoderate broad-spectrum antifungal activities against *C. cladosporioides, C. cucumerinum, Saccharomyces cerevisiae, Aspergillus niger*, and *Rhizopus oryzae*, with MICs of 82.9, 165.8, 165.8, 331.6, and 331.6 μM, respectively (Siriwach et al., 2014). An endophytic *Xylaria* sp. with broad antimicrobial activity was isolated from *Ginkgo biloba* L. Bioactivity-guided fractionation led to the identification of 7-amino-4-methylcoumarin (35) (Liu et al., 2008). Antimicrobial assay showed that 35 inhibited the growth of the tested 13 pathogens including *Penicillium expansum* (MIC, 228.6 μM) and *A. niger* (142.8 μM) (Liu et al., 2008).

### Terpenoids

**FIGURE 3:**
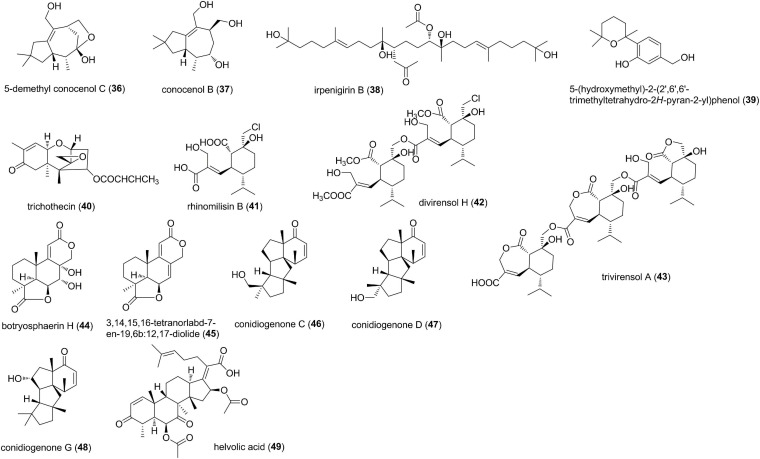
Terpenoids characterized from endophytic fungi (36–49).

Terpenoids produced by fungi are one of the most numerous and structurally diverse secondary metabolites with a wide array of pharmacological properties (Figure 3). The coculture of the phytopathogenic *Nigrospora oryzae* and endophytic *Irpex lacteus* from the same host *Dendrobium officinale* afforded a new tremulane sesquiterpene 5-demethyl conocenol C (36), conocenol B (37), and a new squalene irpenigirin B (38) (Wu et al., 2019). The new compounds 36 and 38 were active against *C. gloeosporioides*, with MICs of 31.7 and 13.4 μM, while 36 showed antifungal activity against *Didymella glomerata* with an MIC of 3.9 μM (Wu et al., 2019). It was also found that the mutually antagonistic relationship between the phytopathogens and endophytes can lead to the production of antibiotics, which inhibit the growth of phytopathogens and hinder certain phytotoxins (Wu et al., 2019). A new natural sesquiterpene 5-(hydroxymethyl)-2-(2′,6′,6′-trimethyltetrahydro-2*H*-pyran-2-yl)phenol (39) was characterized from endophytes belonging to the *Lophodermium* sp., which were isolated from the needles of superior *Pinus strobus* (eastern white pine) trees (Sumarah et al., 2011). 39 was antifungal against the rust *Microbotryum violaceum* with an MIC of 2 μM (Sumarah et al., 2011). *Trichothecium roseum* LZ93, an endophyte from medicinal plant *Maytenus hookeri*, was found to antagonize various phytopathogens *in vitro*. Chemical investigation of this fungal strain afforded a trichothecene, trichothecin (40), with weak to moderate inhibition on phytopathogenic fungi *Typhula incarnate* (MIC, 150.6 μM), *Gaeumannomyces graminis* (MIC, 90.4 μM), *Phytophthora infestans* (MIC, 90.4 μM), *A. solani* (MIC, 15.1 μM), and *Phyricularia oryzae* (MIC, 60.2 μM) (Zhang et al., 2010). A monosesquiterpene rhinomilisin B (41), a new dimeric sesquiterpene divirensol H (42), and an unprecedented trimeric sesquiterpene trivirensol A (43), were purified from *T. virens* FY06, an endophyte derived from *Litchi chinensis* Sonn (Hu et al., 2019). Compounds 41-43 exhibited moderate to potent activities on *Penicillium italicum, F. oxysporum*, *F. graminearum, Colletotrichum musae*, and *C. gloeosporioides*. 41 showed potent inhibitory activity against *C. musae*, with an MIC value of 37.4 μM, which was superior to that of the positive control triadimefon (273.0 μM). Moreover, 42 was more active toward *F. oxysporum, C. gloeosporioides, C. musae, P. italicm*, and *F. graminearum*, which were 8, 8, 3.2, 8, and 24 times as high as those of triadimefon (Hu et al., 2019). Interestingly, as metabolites of the endophytic fungus from *L. chinensis*, all the isolated sesquiterpenes presented potent antifungal activities against *C. gloeosporioides*. The phytopathogenic fungus *C. gloeosporioides* can cause anthracnose in *L. chinensis*, indicating that the metabolites produced by endophytes from its host may play a defensive role by inhibiting invasive phytopathogens. Two tetranorlabdane diterpenoids, botryosphaerin H (44) and 13,14,15,16-tetranorlabd-7-en-19,6β:12,17-diolide (45), were isolated from the endophytic fungus *Botryosphaeria* sp. P483 of the Chinese herbal medicine *Huperzia serrata* (Chen et al., 2015). Compounds 44 and 45 showed strong antifungal activities against *G. graminis, F. moniliforme, F. solani, F. oxysporum* and *Pyricularia oryzae* at 100 μg/disk (Chen et al., 2015). Three diterpenes, conidiogenones C (46), D (47), and G (48), were isolated from an endophytic fungus *Leptosphaeria* sp. XL026 derived from the leaves of *Panax notoginseng* (Chen et al., 2019). Compounds 46 and 48 showed moderate antifungal activity against *Rhizoctonia cerealis*, as well as 47 against *Verticillium dahlia*, with an MIC value of 41.4 μM (Chen et al., 2019). A nordammarane triterpenoid helvolic acid (49) was obtained from *Aspergillus fumigatus*, an endophytic fungus associated with *Melia azedarach* (Li et al., 2012). 49 exhibited broad-spectrum and potent antifungal activities against *B. cinerea, A. solani, A. alternata, C. gloeosporioides, F. solani, F. oxysporum, F. oxysporum*, and *G. saubinettii*, with MIC values of 11.0-88.1 μM (Li et al., 2012).

### Polyketides

**FIGURE 4:**
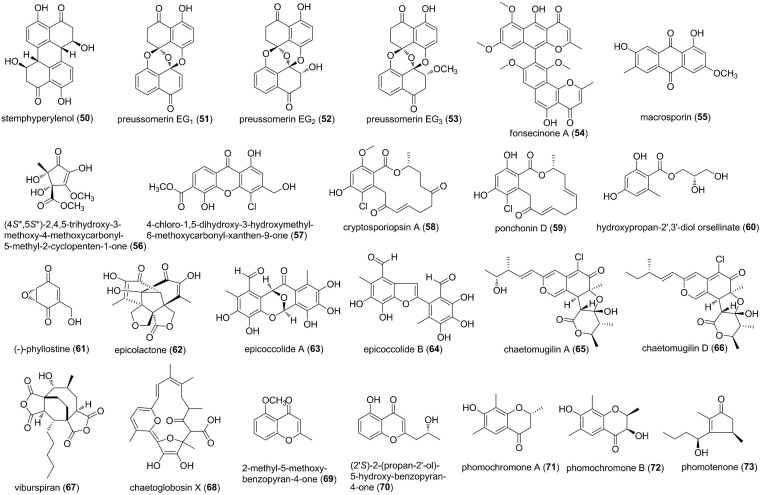
Polyketides characterized from endophytic fungi (50–73).

**FIGURE 5:**
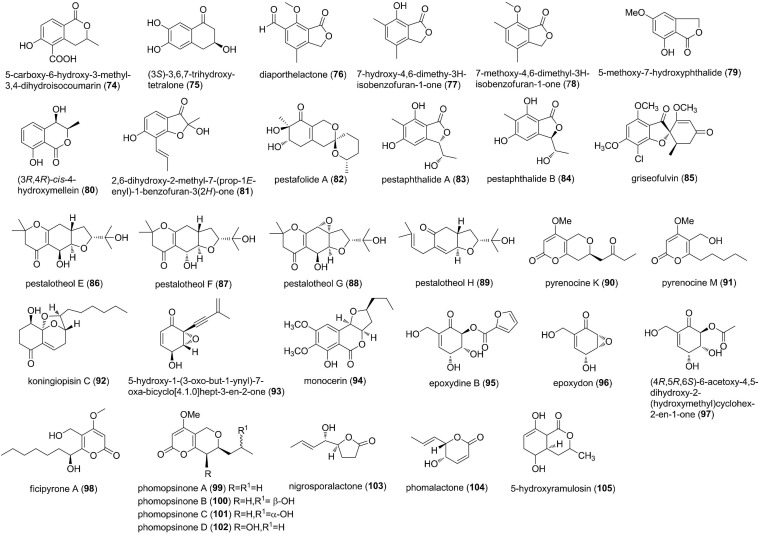
Polyketides characterized from endophytic fungi (continued) (74–105).

**FIGURE 6:**
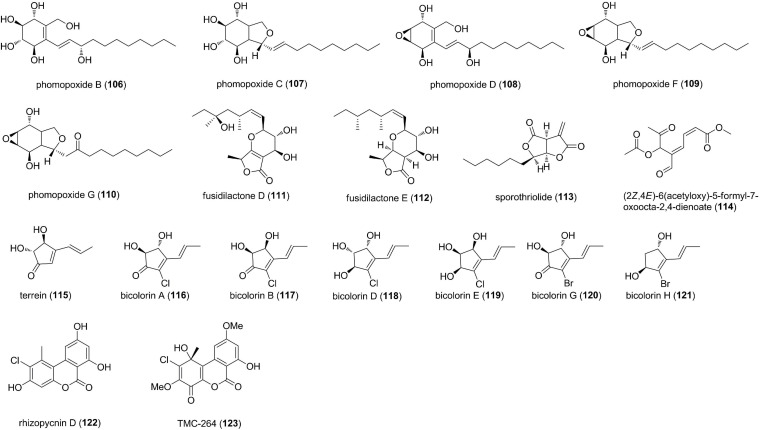
Polyketides characterized from endophytic fungi (continued) (106–123).

Polyketides are a large class of structurally diverse natural products that exhibit a wide range of bioactivities. These metabolites are generally biosynthesized by polyketide synthases, and simultaneously, some of them possess hybrid chemotypes derived from different biosynthetic pathways. In this review, chromones, quinines, macrolides, benzopyrones, unsaturated lactones, and phenols are all classified into polyketides (Figures 4–6). The culture of the fungus *Botryosphaeria dothidea* KJ-1, isolated from the stems of white cedar (*Melia azedarach* L.), yielded a perylenequinone derivative stemphyperylenol (50) (Xiao et al., 2014b). 50 displayed potent antifungal activity against *A. solani* with an MIC of 1.57 μM, compared to the commercially available fungicide carbendazim (Xiao et al., 2014b). Cultivation of the newly discovered fungal strain *Edenia gomezpompae*, an endophyte obtained from the leaves of *Callicarpa acuminata* (Verbenaceae), afforded three new naphthoquinone spiroketals, preussomerins EG_1_ (51), EG_2_ (52), and EG_3_ (53) (Macías-Rubalcava et al., 2008). Spiroketals 51-53 displayed significant growth inhibition against four economically important phytopathogens (*Phythophtora capsici, P. parasitica, F. oxysporum*, and *A. solani*), with IC_50_ values ranging from 57.5 to 447.4 μM (Macías-Rubalcava et al., 2008). Fonsecinone A (54), a dimeric naphtha-γ-pyrone, was characterized from the culture of *Aspergillus* sp. KJ-9, an endophytic fungus residing in the stem bark of *Melia azedarach* (Xiao et al., 2014a). Compound 54 had marked inhibition of *G. saubinetti, Magnaporthe grisea, B. cinerea*, and *A. solani*, with MICs in the range of 6.25-50 μM, which were better than or similar to that of hymexazol (MIC 50 μM) (Xiao et al., 2014a). An anthraquinone, macrosporin (55), was isolated from the mangrove endophytic fungus *Phoma* sp. L28 (Huang et al., 2017). 55 possessed broad-spectrum antifungal activities against *F. oxysporum, F. graminearum, C. musae, P. italic, R. solani*, and *C. gloeosporioides*, with MIC values ranging from 13.2 to 252.1 μM. It should be pointed out that the inhibitory activity of 55 against *F. oxysporum* (MIC = 13.2 μM) was higher than that of the positive control carbendazim (MIC = 32.7 μM) (Huang et al., 2017). Then, from the same host plant (semi-mangrove plant *Myoporum bontioides*), an endophytic fungus *Alternaria* sp. R6 was isolated. A racemic new cyclopentenone derivative, (±)-(4*S*^∗^,5*S*^∗^)-2,4,5-trihydroxy-3-methoxy-4-methoxycarbonyl-5-methyl-2-cyclopenten-1-one (56), and a new xanthone 4-chloro-1,5-dihydroxy-3-hydroxymethyl-6-methoxycarbonyl-xanthen-9-one (57), were characterized from this fungal strain (Wang et al., 2015). In comparison to the positive control triadimefon (MIC 510.64 μM), compounds 56 and 57 exhibited inhibitory activities against *F. graminearum* with MICs of 215.52 and 107.14 μM, respectively, while 57 also showed potent antifungal activity against *C. musae* with an MIC of 214.29 μM (Wang et al., 2015). *Cryptosporiopsis* sp., an endophytic fungus from the medicinal plant *Zanthoxylum leprieurii*, was the source of new polyketides, cryptosporiopsin A (58), ponchonin D (59), hydroxypropan-2′,3′-diol orsellinate (60), and (-)-phyllostine (61) (Talontsi et al., 2012). Compounds 59-61 exhibited motility inhibitory and zoosporicidal activities against *P. viticola* zoospores at 28.6-71.4 μM. Meanwhile, they also displayed mycelial growth of *P. ultimum, A. cochlioides*, and *R. solani* with MICs of 20-40 μg/disk (Talontsi et al., 2012). An endophytic fungus, *Epicoccum* sp. CAFTBO, obtained from the cocoa tree *Theobroma cacao* was found to produce three new unprecedented polyoxygenated polyketides, epicolactone (62), epicoccolides A (63) and B (64) (Talontsi et al., 2013). Compounds 62-64 significantly inhibited the growth of three notorious crop-devastating phytopathogens *P. ultimum, A. cochlioides*, and *R. solani* with MICs of 20-80 μg/disk (Talontsi et al., 2013). Two chlorine-substituted azaphilones, chaetomugilins A (65) and D (66), were isolated from endophytic *C. globosum* of *Panax notoginseng* (Li et al., 2016). Compounds 65 and 66 had moderate activity against *E. nigrum* with the MIC values of 17.8 and 36.9 μM, respectively (Li et al., 2016). Viburspiran (67), a structurally novel maleic anhydride natural products with an additional ethylene bridge, was isolated the fungal endophyte *Cryptosporiopsis* sp. from *Viburnum tinus* (Saleem et al., 2011). 67 was active against *Microbotryum violaceum* and *B. cinerea*, with inhibition radius of 6 and 10 mm at 50 μg substance/filter disk (Saleem et al., 2011). A new macrocyclic metabolite, chaetoglobosin X (68), was isolated from an endophytic fungus *C. globosum* obtained from the medicinal plant *Curcuma wenyujin* (Wang Y. et al., 2012). 68 possessed reasonably potent fungistatic activities on *Exserohilum turcicum, F. oxysporum*, and *Curvularia lunata* with an MIC of 7.5 μM and showed moderate activity against *F. graminearum* and *F. moniliforme* with an MIC of 15.1 μM (Wang Y. et al., 2012). Two benzopyran derivatives, 2-methyl-5-methoxy-benzopyran-4-one (69) and (2′*S*)-2-(propan-2′-ol)-5-hydroxy-benzopyran-4-one (70), were isolated from the isolate of *Curvularia* sp., which was obtained from the leaves of a native plant *Ocotea corymbosa* (Teles et al., 2005). Compounds 69 and 70 exhibited moderate antifungal activity against *C. sphaerospermum* and *C. cladosporioides* with a detection limit of 10 μg (Teles et al., 2005). Two new chromones, phomochromones A and B (71 and 72), and one new natural cyclopentenone derivative, phomotenone (73), was obtained from *Phomopsis* sp., an endophyte isolated from *Cistus monspeliensis* (Ahmed et al., 2011). Compounds 71-73 showed moderate antifungal properties toward *Microbotryum violaceum*, with the radius of the inhibition zone of 8, 5, and 8 mm, respectively, at concentration of 50 μL of 1 mg/mL (Ahmed et al., 2011).

5-carboxy-6-hydroxy-3-methyl-3,4-dihydroisocoumarin (74) was produced from the endophyte *Xylaria* sp., associated with leaves of *Casearia sylvestris* (Chapla et al., 2018). 74 exhibited potent antifungal activities against two phytopathogenic fungi *C. cladosporioides* and *C. sphaerospermum* at 10 μg (Chapla et al., 2018). Bioassay-guided fractionation of the endophytic fungus *Phoma* sp. ZJWCF006 in the Chinese medicinal plant *Arisaema erubescens* afforded a new α-tetralone derivative, (3*S*)-3,6,7-trihydroxy-α-tetralone (75) (Wang L. W. et al., 2012). 75 showed selective growth inhibition against *F. oxysporium* and *R. solani* with EC_50_ values of 2.1 and 0.3 mM, respectively, whereas no obvious activity was observed against *C. gloeosporioides* and *Magnaporthe oryzae* (Wang L. W. et al., 2012). Three known isobenzofuranones including diaporthelactone (76), 7-hydroxy-4,6-dimethy-3*H*-isobenzofuran-1-one (77), and 7-methoxy-4,6-dimethyl-3*H*-isobenzofuran-1-one (78) were obtained from the mangrove endophytic fungus *Phomopsis* sp. A123 from the foliage of *Kandelia candel* (Zhang W. et al., 2014). Compounds 76 and 77 displayed antifungal activity against *A. niger* with MICs of 243 and 485 μM, respectively, while 78 inhibited the growth of *A. alternaria* with an MIC of 500 μM (Zhang W. et al., 2014). Chemical and biological study on an unidentified Ascomycete, an endophyte isolated from *Meliotus dentatus*, led to the isolation of 5-methoxy-7-hydroxyphthalide (79) and (3*R*,4*R*)-*cis*-4-hydroxymellein (80) (Hussain et al., 2009). Compounds 79 and 80 showed antifungal activity against *Microbotryum violaceum* with the radius of zone of inhibition of 7 and 8 mm at a concentration of 50 μL of 1 mg/mL (Hussain et al., 2009). An antifungal strain *Verticillium* sp. from the roots of *Rehmannia glutinosa* yielded 2,6-dihydroxy-2-methyl-7-(prop-1*E*-enyl)-1-benzofuran-3(2*H*)-one (81) (You et al., 2009). 81 clearly inhibited biomass accumulation at a low concentration of 4.4 μM on the pathogens *Septoria* sp. and *Fusarium* sp. The growth of the producing strain *Verticillium* sp. itself was also inhibited to some degree by 81 (You et al., 2009). Pestafolide A (82), a novel reduced spiro azaphilone derivative, and pestaphthalides A (83) and B (84), two new isobenzofuranones, were isolated from *Pestalotiopsis foedan*, an endophyte from an unidentified tree (Ding et al., 2008). 82 displayed antifungal activity against *A. fumigatus*, affording a zone inhibition of 10 mm at 100 μg/disk, whereas 83 and 84 showed activity against *C. albicans* and *G. candidum*, with the zone inhibition of 13 and 11 mm, respectively (Ding et al., 2008). Griseofulvin (85) was produced by *Nigrospora* sp. LLGLM003, an endophytic fungus of the medicinal plant *Moringa oleifera* Lam (Zhao et al., 2012). *In vitro* antifungal assay indicated that 85 displayed potent inhibition of the test eight plant pathogenic fungi; of particular note was the antifungal activity against *B. cinerea* and *Colletotrichum orbiculare* with the EC_50_ of 0.6 and 1.4 μM, respectively (Zhao et al., 2012). Four new metabolites, pestalotheols E-H (86-89), containing a reduced tetrahydro-2*H*-furo[3,2-*g*]chromene unit, were isolated from a fungal endophyte, an unidentified Ascomycete from the tree *Arbutus unedo* (Qin et al., 2011). Compounds 86-89 showed antifungal activity against *Microbotryum violaceum*, with the radius of the inhibition zone of 6, 8, 6, and 7 mm, respectively, at 50 μg of test substance/test filter disk (Qin et al., 2011). Phytochemical studies of the active constituents of the endophytic fungus *Phomopsis* sp. led to the isolation of two new pyrenocine derivatives pyrenocine K (90) and M (91) (Hussain et al., 2012a). Both compounds also showed antifungal activity against *M. violaceum* in an agar diffusion assay, with inhibition zone of 5 mm at a concentration of 0.05 mg (Hussain et al., 2012a). A new fungal polyketide, koningiopisin C (92), was characterized from the culture broth of the fungus *Trichoderma koningiopsis* YIM PH 30002 from the medicinal plant *Panax notoginseng* (Liu et al., 2016). 92 exhibited antifungal activity against *Plectosphaerella cucumerina* with an MIC of 57.1 μM (Liu et al., 2016). The endophyte *Drechslera* sp. strain 678 was isolated from the roots of an Australian native grass *Neurachne alopecuroidea*, which demonstrated efficacy against several plant pathogens (d’Errico et al., 2020). Metabolomic analysis revealed the presence of two major bioactive metabolites, an alkynyl substituted epoxycyclohexenone derivative (5-hydroxy-1-(3-oxo-but-1-ynyl)-7-oxa-bicyclo[4.1.0]hept-3-en-2-one) (93) and monocerin (94). 93 and 94 were active against *B. cinerea* and *S. sclerotiorum* at 10 and 100 μg (d’Errico et al., 2020). A new epoxydon derivate epoxydine B (95), along with two related metabolites, epoxydon (96) and (4*R*,5*R*,6*S*)-6-acetoxy-4,5-dihydroxy-2-(hydroxymethyl)cyclohex-2-en-1-one (97), were obtained from an endophytic fungus, *Phoma* sp., isolated from the plant *Salsola oppostifolia* (Qin et al., 2010). Compounds 95-97 were biologically active exhibiting antifungal activity against *M. violaceum* at a concentration of 0.05 mg (Qin et al., 2010). A new α-pyrone (2*H*-pyran-2-one), ficipyrone A (98), was isolated from solid cultures of the plant endophytic fungus *Pestalotiopsis fici* from the tea plant *Camellia sinensis* (Liu et al., 2013). 98 displayed weak antifungal activity against *Gibberella zeae*, with an IC_50_ value of 15.9 μM, compared with the positive control ketoconazole (IC_50_ 6.02 μM) (Liu et al., 2013). Structural elucidation of the metabolites produced by the endophytic *Phomopsis* sp. revealed four new α-pyrone derivatives, phomopsinones A-D (99-102) (Hussain et al., 2012b). 99 showed potent antifungal activity against *B. cinerea* (inhibition zone of 17 mm), *P. oryzae* (25 mm), and *Septoria tritici* (20 mm), while 102 was active against *B. cinerea* (10 mm) and *S. tritici* (10 mm) (Hussain et al., 2012b). The fermentation of the endophytic fungus *Nigrospora* sp. YB-141 isolated from *A. indica* yielded two lactones, nigrosporalactone (103) and phomalactone (104) (Wu et al., 2009). Compounds 103 and 104 were active against *B. cinerea* with MIC values of 200.3 and 405.8 μM (Wu et al., 2009). Preliminary screening for antimicrobial activity of the endophytic fungi from *Cinnamomum mollisimum* yielded a polyketide, 5-hydroxyramulosin (105), which was identified as the major constituent of the bioactive fungal extracts (Santiago et al., 2012). 105 inhibited the fungal pathogen *A. niger* with the IC_50_ value of 7.9 μM (Santiago et al., 2012).

Five new polyoxygenated cyclohexenoids, phomopoxides B (106), C (107), D (108), F (109), and G (110), were isolated from an endophytic fungal strain *Phomopsis* sp. YE3250 from the medicinal plant *Paeonia delavayi* (Huang et al., 2018). Compounds 106-110 were active against five pathogenic fungi (*C. albicans, A. niger, P. oryzae, F. avenaceum*, and *Hormodendrum compactum*) with MICs of 46.5-372.1 μM (Huang et al., 2018). Investigatation of the metabolites produced by the endophytic *Fusidium* sp., isolated from the leaves of *Mentha arvensis*, found two new bicyclic fusidilactones D (111) and E (112) (Qin et al., 2009). Both compounds had only moderate activity toward *Microbotryum violaceum*, with the radius of the inhibition zone of 7 and 10 mm, respectively (Qin et al., 2009). Through screening antifungal activity of endophytic fungi and subsequent bioassay-guided fractionation, sporothriolide (113) was isolated from the selected endophyte *Nodulisporium* sp. A21 in *Ginkgo biloba* (Cao et al., 2016). 113 was validated to be potently antifungal against the mycelia growth of *R. solani, S. sclerotiorum* and inhibit conidium germination of *M. oryzae in vitro* and *in vivo* (Cao et al., 2016). The screening of fungal extracts of *Lophodermium* sp. isolated from *P. strobus* resulted in the discovery of a new aliphatic polyketide, (2*Z*,4*E*)-6(acetyloxy)-5-formyl-7-oxoocta-2,4-dienoate (114) (Sumarah et al., 2011). 114 was antifungal against *M. violaceum* with MIC of 2 μM (Sumarah et al., 2011). Terrein (115) was isolated from the endophytic fungus *Aspergillus terreus* JAS-2 associated with medicinal plant *Achyranthus aspera* (Goutam et al., 2017). In antifungal assay, 10 μg/μL concentration of 115 showed inhibition of *Bipolaris Sorokiniana* (57.14%), *A. flavus* (52.5%), and *A. alternata* (91.25%) as compared to control (Goutam et al., 2017). Six new halogenated cyclopentenones, including four chlorinated, bicolorins A (116), B (117), D (118), and E (119), and two brominated bicolorins G (120) and H (121), were isolated from the endophytic fungus *Saccharicola bicolor* of *Bergenia purpurascens* (Zhao et al., 2020). Compounds 116-121 possessed weak to moderate activity against five pathogenic fungi-*Uromyces viciae-fabae, Pythium dissimile, G. zeae, A. niger*, and *S. sclerotiorum*, with MICs of 26.8-380.9 μM. 117 and 118, in particular, exhibited moderate activity against *P. dissimile* with the MICs of 33.0 and 44.7 μM, respectively, compared with the positive control cycloheximide (MIC 30.6 μM). Additionally, 118 was proven to be potently antifungal against *S. sclerotiorum in vivo*, indicating its potential as a candidate of new natural fungicides (Zhao et al., 2020). A new dibenzo-α-pyrone rhizopycnin D (122) and a known congener TMC-264 (123) were isolated from the endophytic fungus *Rhizopycnis vagum* Nitaf22 obtained from *Nicotiana tabacum* (Lai et al., 2016). Both compounds inhibited the spore germination of *M. oryzae* with IC_50_ values of 33.9 and 34.1 μM, respectively (Lai et al., 2016).

### Miscellaneous Compounds

**FIGURE 7:**
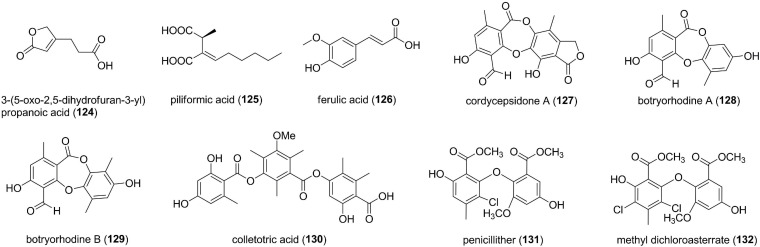
Miscellaneous compounds characterized from endophytic fungi (124–132).

A new furan derivative named 3-(5-oxo-2,5-dihydrofuran-3-yl) propanoic acid (124) was isolated from an endophytic *Aspergillus tubingensis* of *Decaisnea insignis* (Figure 7; Yang X. F. et al., 2019). 124 exhibited potent antifungal activity against *F. graminearum* with MIC value of 102.6 μM (Yang X. F. et al., 2019). Piliformic acid (125), derived from octanoate that originates from a fatty acid synthase, was obtained from endophytic fungi *Xylaria* sp., which were isolated from leaves of guarana plant (Elias et al., 2018). 125 had antifungal activity against *C. gloeosporioides* with an MIC of 2.92 μM (Elias et al., 2018). The endophytic fungus *Aspergillus* sp. from *Moringa oleifera* produced one phenolic acid, ferulic acid (126), which showed a weak antifungal activity at 500 μg/mL against *A. niger* with an inhibition zone diameter of 2 mm (Abonyi et al., 2018). Cordycepsidone A (127), a depsidone metabolite, was isolated from *Cordyceps dipterigena*, an endophytic fungus from *Desmotes incomparabilis* antagonistic to the phytopathogen *Gibberella fujikuroi* (Varughese et al., 2012). 127 showed a moderate to potent growth inhibitory activity against *G. fujikuroi* (MIC, 23.3 μM) and *Pythium ultimum* (MIC, 3.4 μM) (Varughese et al., 2012). An endophyte *Botryosphaeria rhodina* was isolated from the stems of the medicinal plant *Bidens pilosa* and was chosen for further chemical study due to its potent antifungal effects (Abdou et al., 2010). Bioactivity-guided fractionation of this strain yielded two new depsidones, botryorhodine A (128) and B (129), which were significantly active against *A. terreus* with MICs of 26.03 and 49.70 μM (Abdou et al., 2010). A new tridepside, colletotric acid (130), was characterized from *C. gloeosporioides*, an endophytic fungus colonized inside the stem of *Artemisia mongolica* (Zou et al., 2000). 130 inhibited the growth of the crop pathogenic fungus *Helminthosporium sativum* with an MIC of 95.4 μM (Zou et al., 2000). From an isolate of *Aspergillus* from a healthy plant of oilseed rape (*Brassica napus*), two chlorinated diphenyl ethers, penicillither (131) and methyl dichloroasterrate (132), were characterized (Qin et al., 2019). Both of them inhibited four plant pathogenic fungi (*B. cinerea, M. fructicola, S. sclerotiorum*, and *S. trifoliorum*) with the EC_50s_ of 21.7-151.2 μM (Qin et al., 2019).

## Potential Use in the Development of Biopesticides

As mentioned above, biologically active and structurally diverse fungal metabolites constitute a rich resource for drugs and pesticides discovery. These recently discovered metabolites 1-132, which possessed extensive chemical skeletons, exhibited moderate to potent anti-phytopathogenic activities. Therefore, some of them might have the potential use in the development of new biopesticides. Especially, based on these potential compounds, a series of novel derivatives with agricultural and pharmaceutical importance were designed and synthesized.

### Griseofulvin

**FIGURE 8:**
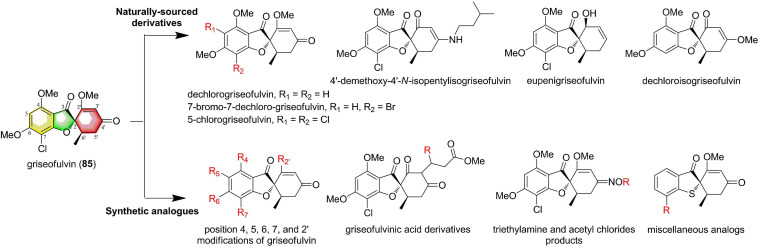
Selected structures of naturally sourced griseofulvin derivatives and synthetic analogs.

Griseofulvin (85), a secondary metabolite possessing spirocyclic benzofuran-3-one skeleton, was initially isolated from the fungus *Penicillium griseofulvum* in 1939 by Oxford et al. (Petersen et al., 2014). Then, this polyketide has also been found to be produced by several Ascomycetes including *Penicillium* sp., *Aspergillus* sp., and *Xylaria* sp. (Zhao et al., 2012). Griseofulvin has a rich chemical diversity, and until now, more than 400 griseofulvin analogs have been isolated and synthesized (Figure 8; Petersen et al., 2014). Griseofulvin was one of the first antifungal metabolic products in filamentous fungi, offered *in vitro* fungistatic effect against dermatomycoses. Recently, it has gained renewed attention due to many reports of antifungal properties against plant pathogenic fungi. Zhao et al. reported that griseofulvin, produced by an endophyte *Nigrospora* sp., displayed clear growth inhibition of the test eight plant pathogenic fungi (*B. cinerea, Colletotrichum orbiculare, F. oxysporum f.sp. cucumerinum, F. oxysporum f.sp. melonis, Pestalotia diospyri, Pythium ultimum, R. solani*, and *S. sclerotiorum*) (Zhao et al., 2012). Among them, it exhibited potent activity against *B. cinerea* and *C. orbiculare* with the EC_50_ values of 0.6 and 1.4 μM, respectively (Zhao et al., 2012). It should be pointed out that, its dechlorinated derivative, dechlorogriseofulvin, only showed weak activity, indicating that the chlorine played a decisive role in the antifungal activity. Tang et al. reported that griseofulvin isolated from *Penicillium brasilianum* displayed strong inhibitory effect on the growth of *A. solani* with an MIC of 3.13 μM (Tang et al., 2015). These impressive activities make this compound suitable candidate for biopesticide discovery and trigger the following synthesis studies, including semisynthesis from griseofulvin and *de novo* synthesis.

Bai et al. designed and synthesized 22 griseofulvin derivatives from commercially available griseofulvin (Bai et al., 2019). *In vitro* antifungal assay indicated that griseofulvin and its derivatives possessed remarkable activities against five phytopathogenic fungi (*Cytospora sp., C. gloeosporioides, B. cinerea, A. solani*, and *F. solani*) (Bai et al., 2019). Of significance was that, compounds numbered 6a-6f were found to have significant potential, which were superior to commercial fungicides hymexazol and thiophanate-methyl. The three-dimensional quantitative structure-activity relationship analysis revealed that the modification of the 4’ position, for example, the suitable bulky and electronegative acyl-substituted groups at the 4’ position, can significantly improve the antifungal activity, even up to 10-fold higher than inhibitory effect of the parent compound griseofulvin (Bai et al., 2019). Kartsev et al. carried out the synthetic studies of griseofulvin derivatives (Kartsev et al., 2019). As a result, a total of 42 new griseofulvin derivatives were designed and synthesized. These newly synthesized griseofulvin derivatives exhibited potent antifungal activity against *A. niger, A. ochraceus, A. fumigatus, A. versicolor, Penicillium funiculosum, P. ochrochloron, P. verucosum* var. *cyclopium, Trichoderma viride*. All compounds showed higher activity than the commercial antifungal drugs ketoconazole (7-42 times) and bifonazole (3-16 fold) (Kartsev et al., 2019). Interestingly, the synthesized compounds were more active than the parent compound griseofulvin (up to 4 times). Therefore, in conclusion, griseofulvin especially its derivatives can be further used for the development of new agricultural fungicides.

### Trichothecene

**FIGURE 9:**
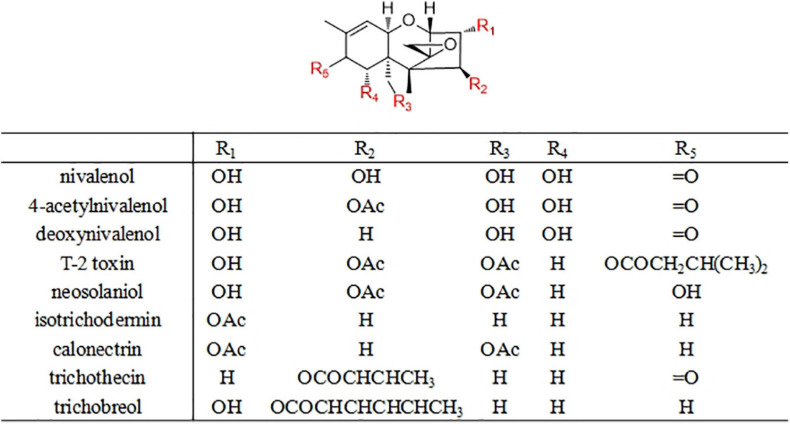
Representative antifungal trichothecene sesquiterpenes derivatives.

Trichothecenes, a group of sesquiterpene-based fungal metabolites with a common tricyclic 12,13-epoxytrichothec-9-ene core, are found to be produced by various microorganisms such as Fusarium sp., Myrothecium sp., Spicellum sp., Stachybotrys sp., Cephalosporium sp., Trichoderma sp., and Trichothecium sp. Based on the substitution pattern, trichothecenes are classified into different families of nivalenols, neosolaniols, isotrichodermins, calonectrins, trichothecins, and trichobreols (Figure 9; Takahashi-Ando et al., 2020). Trichothecenes are well-known as mycotoxins, causing significant negative effects on agriculture and human health. Initial study of trichothecenes was focused on the phytotoxicity and mammalian intoxications, and later emphasis was on exploring their complementary bioactivities. Some related compounds exhibited potent antitumor effect and have been used for clinical trials (Li et al., 2017). Meanwhile, trichothecenes showed varied role in the field of agriculture and may act as bio-control agents, strengthen the defense system of the plants against pathogens (Kumari et al., 2016). Previous structure–activity relationships study revealed that a slight modification of trichothecenes could dramatically decrease the toxicity but still retain its bioactivity (Li et al., 2017). In this case, there seems to be a special significance to search for new trichothecenes with agricultural applications.

Yamazaki et al. reported three new antifungal trichothecenes, trichobreols A-C, from the NaI-containing fermentation of the marine-derived Trichoderma cf. brevicompactum (Yamazaki et al., 2020a). Then, from the same fungus, another two new trichothecenes, trichobreols D and E, were obtained (Yamazaki et al., 2020b). Trichobreols showed good antifungal activity, especially toward yeast-like pathogenic fungi. Moreover, five semisynthetic derivatives were prepared from trichobreol A to evaluate the structure–activity relationship of antifungal trichothecenes. The results indicated that the substituents at C-3 and C-4 positions were responsible for the potency of antifungal activity (Yamazaki et al., 2020b). Li et al. reported three new macrocyclic trichothecenes possessing rare 6’-ketal moieties, roridoxins A-C, from the insect-associated fungus Myrothecium roridum (Li et al., 2019). Roridoxins A and C were found to possess potent antifungal activity against Alternaria tenuissima, A. niger, Pyricularia grisea, and F. oxysporum (Li et al., 2019). These findings suggested that trichothecenes are also promising leads which are applicable for the development of new agrochemicals.

## Conclusion and Future Perspectives

**FIGURE 10:**
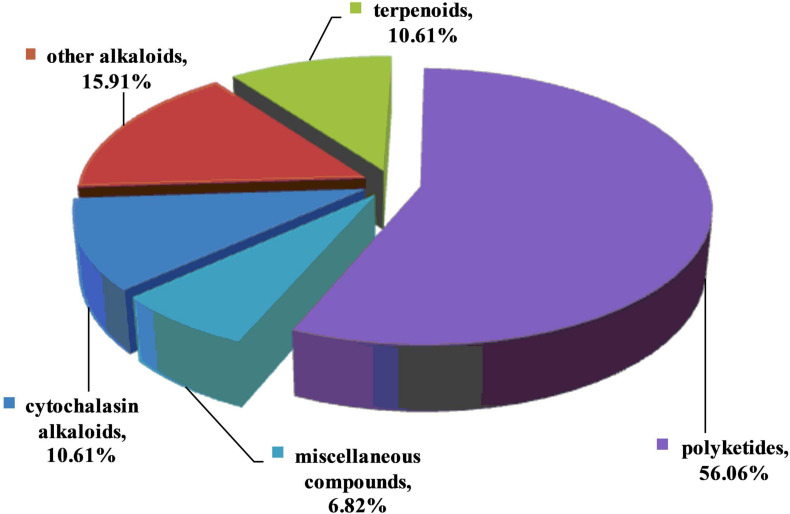
Percentage distributions of antifungal metabolites based on their putative biogenetic origin.

Fungal endophytes, which are ubiquitous in plants and symbiotic with their hosts, are well-known for producing a variety of antimicrobial metabolites and enhancing plant resistance to pathogens and pests. These bioactive metabolites play a defensive role in protecting the host plants against pathogenic attacks. Therefore, antibiotic metabolites from the endophytes have the potential to be applied as agrochemicals to control pathogens. This review summarizes the structural/biogenetic types of 132 antifungal metabolites isolated from fungal endophytes in the past two decades. These present metabolites possess diverse chemical structures. Based on their putative biogenetic origin, they were classified into alkaloids (including 1-14 for cytochalasins and 15-35 for other alkaloids), terpenoids (36-49), polyketides (50-123), and other miscellaneous compounds (124-132). It is worth mentioning that the structural classifications based on biogenetic categories are somewhat arbitrary, as many compounds are derived from mixed biosynthetic pathways. Taking compounds 86-89 as an example, they are new members of the chromenone-type of metabolites biogenetically derived from isoprenoids and a polyketide. As shown in Figure 10, among the 132 active metabolites, approximately 56% were polyketides. Molecules grouped as polyketides are significant in natural products research due to their biosynthetic complexity and high value in pharmaceutical and/or agrochemical industries. This finding revealed that polyketides are the most potential classes for the discovery of novel antifungal lead compounds. It should be pointed out that we divided cytochalasins into an individual group, since this kind of compounds constitutes 10.6% of the metabolites reported, nearly as many as terpenoids (10.6%) and other alkaloids (15.9%). Cytochalasins are a diverse group of fungal polyketide synthase-non-ribosomal peptide synthetase (PKS-NRPS) hybrid metabolites. This class of compounds are worthy of particular attention and may be applied in the field of bio-pesticides.

**FIGURE 11:**
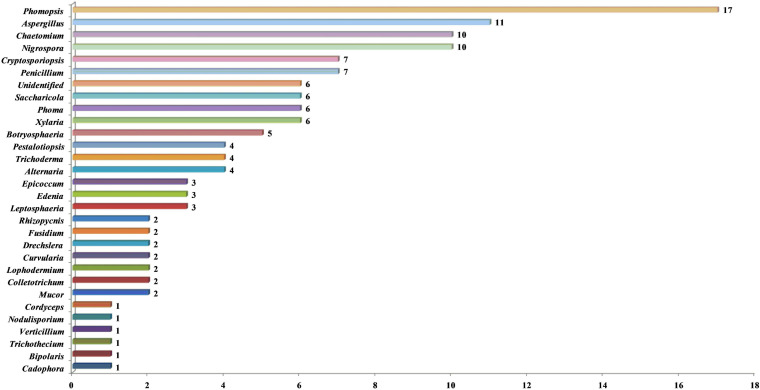
Antifungal metabolites characterized from various producing strains.

It is well-known that the endophytic fungi from terrestrial plants are a treasure house of bioactive secondary metabolites. Moreover, marine-derived endophytic fungi have also been considered as a non-negligible resource to search for antifungal lead compounds. In this review, compounds 4, 10-12, 55-57, and 76-78 were isolated from marine mangrove-associated endophytic fungi. The marine environment is quite different from the terrestrial environment, which indicates that marine-derived endophytic fungi may possess unique metabolic pathways to produce interesting antifungal compounds with novel structures. As for the producing strains, the fungal genera *Phomopsis*, *Aspergillus, Chaetomium*, and *Nigrospora* are predominant genera as producers of these antifungal metabolites, with 17, 11, 10, and 10 compounds described, respectively (Figure 11). As shown in Figure 11, these metabolites are scattered across a variety of fungi belonging to 29 various genera, including some rare species. Among them, *C. globosum* is a creative species known for making a large number of exclusive and structurally significant bioactive chaetoglobosins. The antifungal activity of these metabolites against the phytopathogenic fungi indicated that the endophytes could protect their host plants by producing bioactive molecules, which may be toxic or even lethal to phytopathogens and highlighted the potential of endophytic fungi in producing valuable metabolites. Moreover, a chemical interaction between the endophytes and the host plants, which produces metabolites as chemical defense compounds, needs further investigation.

**FIGURE 12:**
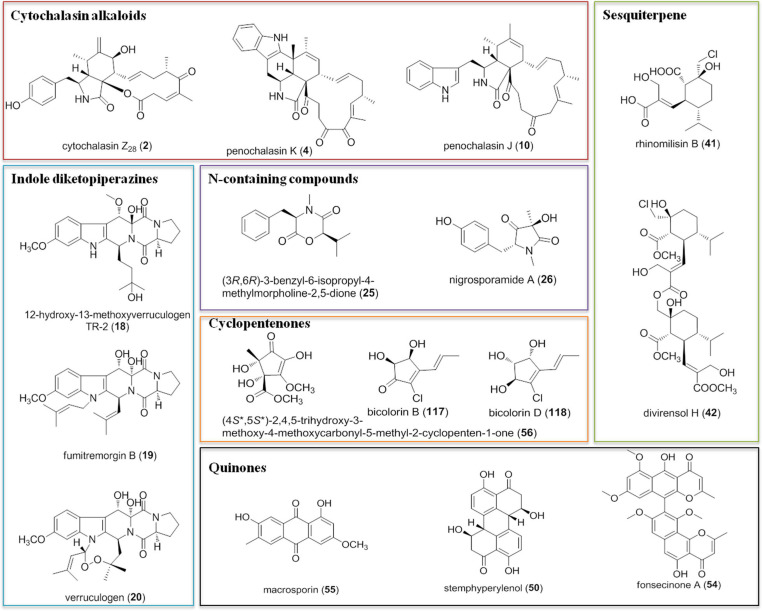
Potential candidates of new natural fungicides.

Most importantly, among the 132 antifungal metabolites presented in this review, some of them not only possessed intriguing chemical structures but also showed novel antifungal activities comparable to those of widely used chemical pesticides. These include cytochalasin alkaloids cytochalasin Z_28_ (2), penochalasin K (4), and penochalasin J (10), indole diketopiperazines 12β-hydroxy-13α-methoxyverruculogen TR-2 (18), fumitremorgin B (19), and verruculogen (20), N-containing compounds (3*R*,6*R*)-3-benzyl-6-isopropyl-4-methylmorpholine-2,5-dione (25) and nigrosporamide A (26), sesquiterpenes rhinomilisin B (41) and divirensol H (42), quinones stemphyperylenol (50), fonsecinone A (54), and macrosporin (55), cyclopentenones (4*S*^∗^,5*S*^∗^)-2,4,5-trihydroxy-3-methoxy-4-methoxycarbonyl-5-methyl-2-cyclopenten-1-one (56), bicolorin B (117), and D (118) (Figure 12). The above mentioned compounds showed potent (or significant) antifungal activities compared to those of positive controls (usually chemical pesticides such as difenoconazole, hymexazol, carbendazim, and triadimefon), which indicates that they could be used as potential alternatives to traditional pesticides.

Overall, endophytes are considered to be a treasure house of antifungal metabolites. This review summarizes 132 metabolites with moderate to potent antifungal activities isolated from fungal endophytes. We also list some “star molecules” such as griseofulvin and its derivatives that possess high potential as candidates of new natural fungicides herein. It is believed that in the near future, research on antifungal metabolites of endophytic fungi will become more prolific and be beneficial for the development of new agrochemicals.

## Author Contributions

KX and X-QL performed the literature material’s collection and reorganization. PZ and D-LZ wrote the manuscript. All authors reviewed the manuscript.

## Conflict of Interest

The authors declare that the research was conducted in the absence of any commercial or financial relationships that could be construed as a potential conflict of interest.

## Supplementary Material

Supplementary Table 1 The producing strain, environment source, and antifungal activities of compounds 1–132.
