# Supplementary material for: Antifungal Secondary Metabolites Produced by the Fungal Endophytes: Chemical Diversity and Potential Use in the Development of Biopesticides
Source: Front Microbiol. 2021 Jun 21;12:689527. doi: 10.3389/fmicb.2021.689527 (PMC8255633; doi:10.3389/fmicb.2021.689527)
Supplement: Supplementary Table 1 — The producing strain, environment source, and antifungal activities of compounds 1–132. [file Table_1.docx]

*Supplementary Material*

Antifungal Secondary Metabolites Produced by the Fungal Endophytes: Chemical Diversity and Potential Use in the Development of Biopesticides

Kuo Xu^1†^, Xiu-Qi Li^1†^, Dong-Lin Zhao^1^*, and Peng Zhang^1^*

^1^Tobacco Research Institute of Chinese Academy of Agricultural Sciences, Qingdao 266101, People’s Republic of China

***Correspondence:**Peng Zhang
zhangpeng@caas.cn

Dong-Lin Zhao
zhaodonglin@caas.cn

^†^These authors contributed equally to this work.

Keywords: Fungal endophytes, Secondary metabolites, Chemical diversity, Phytopathogenic fungi, Antifungal activities, Biopesticides

**Table S1.** The producing strain, environment source, and antifungal activities of compounds **1**–**132**

| **Compounds** | **Producing Strain** | **Plant Source** | **Antifungal Activity** | **Reference** |
| --- | --- | --- | --- | --- |
| cytochalasin D (**1**) | *Xylaria* sp. | From leaves of guarana plant with anthracnose symptoms (necrotic lesions), collected at Manaus-AM (03°22′5) and Maués-AM (57°42′0). | Compound **1** was active against *C. gloeosporioides* with an MIC of 2.46 mM. | [Elias et al., 2018] |
| cytochalasin Z_28_ (**2**) | *Xylaria* sp. | From the healthy leaves of *Toona sinensis*, collected in Yangling, Shaanxi province, China. | Compound **2** showed potent fungicidal effect (MIC = 12.5 μM) against *G. saubinetti*. | [Zhang et al., 2014] |
| rosellichalasin (**3**) | *Aspergillus capensis* CanS‑34A | From a healthy plant of oilseed rape (*Brassica napus*), collected in Wuhan city, Hubei Province of central China (30°28′N, 114°21′E). | Compound **3** inhibited four fungi (*B. cinerea*, *Monilinia fructicola*, *S. sclerotiorum*, and *S. trifoliorum*) with the EC_50_s ranging from 36.8 to 87.1 μM. | [Qin et al., 2019] |
| penochalasin K (**4**) | *Penicillium chrysogenum* V11 | From the vein of *Myoporum bontioides* A. Gray in Leizhou Peninsula, China. | Compound **4** displayed potent inhibitory activities against *C. gloeosporioides* (MIC 6.13 μM) and *R. solani* (MIC 12.26 μM). | [Zhu et al., 2017] |
| chaetoglobosins A (**5**), B (**6**), E (**7**), F (**8**), and penochalasin G (**9**) | *Chaetomium globosum* | From the seeds of *Panax notoginseng* that was collected at the Wenshan, Yunnan, China. | Compounds **5**−**9** showed moderate to potent inhibitory activities against *Phoma herbarum* and *E. nigrum*. | [Li et al., 2016] |
| penochalasin J (**10**), chaetoglobosin C (**11**), and armochaetoglobosin I (**12**) | *Penicillium chrysogenum* V11 | From the vein of *Myoporum bontioides* A. Gray in Leizhou Peninsula. | Compound **10** potently inhibited *C. gloeosporioides* with an MIC of 25.08 μM, while **11** and **12** potently inhibited *R. solani*. | [Huang et al., 2016] |
| chaetoglobosins V (**13**), G (**14**) | *Chaetomium globosum* | From the leaves of *Ginkgo biloba* tree. | Both **13** and **14** exhibited potently antifungal activity against *A. solani*, with an MIC of 47.3 μM. | [Xue et al., 2012] |
| 14-hydroxyterezine D (**15**), terezine E (**16**) | *Mucor* sp. | From the medicinal plant *Centaurea stoebe* growing in Idaho, USA. | Both **15** and **16** exerted weak antifungal efficacy against *A. terreus*, with MICs of 127.8 and 111.2 μM, respectively. | [Abdou et al., 2018] |
| 2-phenylethyl 1*H*-indol-3-yl-acetate (**17**) | *Colletotrichum gloeosporioides* | From the leaves of *Michelia champaca* | Compound **17** exhibited potent activity against *C. cladosporioides* and *C. sphaerospermum* | [Chapla et al., 2014] |
| 12β-hydroxy-13α-methoxyverruculogen TR-2 (**18**), fumitremorgin B (**19**), verruculogen (**20**) | *Aspergillus fumigates* LN-4 | From the healthy stem bark of *Melia azedarach* L., which was collected at Yangling, Shaanxi province, China. | Compounds **18**−**20** showed potent activities against eight pathogens (MICs = 13.7−100 μM). | [Li et al., 2012] |
| 3-O-methylviridicatin (**21**), viridicatol (**22**), 5-hydroxy-8-methoxy-4-pheny lisoquinolin-1(2*H*)-one (**23**) | *Penicillium* sp. R22 | From the root of *Nerium indicum*, collected from Qinling Mountain, Shaanxi Province, China. | Compounds **21**−**23** exhibited weak to moderate antifungal activity against *A. brassicae*, *A. alternata*, and *V. mali*. | [Ma et al., 2018] |
| atransfusarin (**24**), (3*R*,6*R*)-3-benzyl-6-isopropyl-4-methylmorpholine-2,5-dione (**25**) | *Alternaria atrans* MP-7 | From the healthy leaves of *Psidium guajava*, collected from Nanning city, Guangxi Province of China. | Compound **24** exerted weak activity against *B. cinerea* and *A. solani*, while **25** exhibited remarkable activity against *A. solani*, *C. gloeosporioides*, and *P. grisea*. | [Yang et al., 2019] |
| nigrosporamide A (**26**) | *Nigrospora sphaerica* ZMT05 | From *Oxya chinensis* Thunberg collected from Guangzhou, China. | Compound **26** exhibited potent activity against *C. gloeosporioides* with an MIC value of 25.14 μM. | [Zhu et al., 2017] |
| (+)-flavipucine (**27**) | *Phoma* sp. | From leaves of *Salsola oppositifolia*, growing near the waterfront of the Atlantic Ocean, Valle Gran Rey, Gomera, Spain. | Compound **27** showed strong inhibition down to 7.81 ppm against *Phytophthora infestans* and down to 31.3 ppm against *Septoria tritici*. | [Loesgen et al., 2011] |
| cyclo-(L-Ile-L-Leu-L-Leu-L-Leu-L-Leu) (**28**), cyclo-(L-Phe-L-Leu-L-Leu-L-Leu-L-Leu) (**29**) | *Cryptosporiopsis* sp. | From leaves and branches of *Zanthoxylum leprieurii* (Rutaceae), collected at Mont Kala near Yaoundé, Cameroon. | Compound **28** and **29** exhibited motility inhibition of *P. viticola* zoospores, and also inhibited *P. ultimum*, *A. cochlioides*, and *R. solani*. | [Talontsi et al., 2012] |
| cercosporamide (**30**) | *Cadophora orchidicola* | From leaves of *Kalimeris indica*, collected in LongQuan County, Zhejiang Province, China. | Compound **30** had potent growth inhibition against *Pestalotia diospyri*, *B. cinerea*, *F. oxysporum*, *Sclerotium rolfsii* and *P. digitatum*. | [Wang et al., 2019] |
| solanapyrones N (**31**), solanapyrones O (**32**), solanapyrone C (**33**) | *Nigrospora* sp. YB-141 | From stems of *Azadirachta indica* collected in Yuanjiang County, Yunnan Province, China. | Compounds **31−33** were active against *B. cinerea* and *P. islandicum*. | [Wu et al., 2009] |
| bipolamide B (**34**) | *Bipolaris* sp. MU34 | From leaves of *Gynura hispida* Thwaites obtained at the botanical garden in Mahidol University, Bangkok, Thailand. | Compound **34** had moderate activity against *C. cladosporioides*, *C. cucumerinum*, *Saccharomyces cerevisiae*, *A. niger*, and *Rhisopus oryzae*. | [Siriwach et al., 2014] |
| 7-amino-4-methylcoumarin (**35**) | *Xylaria* sp.YX-28 | From twigs of *Ginkgo biloba* L., collected in Jiangsu and Shandong provinces, China. | Compound **35** had potent activities against *P. expansum* (MIC, 228.6 μM) and *A. niger* (142.8 μM). | [Liu et al., 2008] |
| 5-demethyl conocenol C (**36**), conocenol B (**37**), irpenigirin B (**38**) | *Nigrospora oryzae* and *Irpex lacteus* | From seeds of *Dendrobium officinale* collected from Wenshan City in Yunnan Province, China. | Compounds **36** and **38** were active against *C. gloeosporioides* (MIC, 8 μg/mL), and **36** showed activity against *Didymella glomerata* (1 μg/mL). | [Wu et al., 2019] |
| 5-(hydroxymethyl)-2-(2',6',6'-trimethyltetrahydro-2*H*-pyran-2-yl)phenol (**39**) | *Lophodermium* sp. | From the needles of superior *Pinus strobus* (eastern white pine) trees originating in New Brunswick, Canada. | Compound **39** was antifungal against the rust *Microbotryum violaceum* with MIC of 2 μM. | [Sumarah et al., 2011] |
| trichothecin (**40**) | *Trichothecium roseum* LZ93 | From stems of *Maytenus hookeri* Loes, collected at Xishuangbanna, Yunnan, China. | Compound **40** showed weak to moderate inhibition against *Typhula incarnate*, *G. graminis*, *P. infestans*, *A. solani*, *Phyricularia oryzae* (MICs 15.1−150.6 μM). | [Zhang et al., 2010] |
| rhinomilisin B (**41**), divirensol H (**42**), trivirensol A (**43**) | *Trichoderma virens* FY06 | From the root of *Litchi chinensis* Sonn. | Compounds **41−43** exhibited moderate to potent activities on *F. oxysporum*, *C. gloeosporioides*, *C. musae*, *P. italicm*, and *F. graminearum* (MICs, 37.4 μM). | [Hu et al., 2019] |
| botryosphaerin H (**44**), 13,14,15,16-tetranorlabd-7-en-19,6β:12,17-diolide (**45**) | *Botryosphaeria* sp. P483 | From *Huperzia serrata* (Thunb.) Trev., collected in Xichou County, Yunnan Province, China. | Compounds **44** and **45** showed potent activities against *G. graminis*, *F. moniliforme*, *F. solani*, *F. oxysporum* and *P. oryzae* at 100 μg/disk | [Chen et al., 2015] |
| conidiogenone C (**46**), conidiogenone D (**47**), conidiogenone G (**48**) | *Leptosphaeria* sp. XL026 | From the leaves of *Panax notoginseng* which was collected from Shijiazhuang, Hebei province, China. | Compounds **46** and **48** displayed moderate activity against *R. cerealis*, as well as **47** against *V. dahliae* with an MIC value of 41.4 μM. | [Chen et al., 2019] |
| helvolic acid (**49**) | *Aspergillus fumigates* LN-4 | From the healthy stem bark of *Melia azedarach* L. collected at Yangling, Shaanxi, China. | Compounds **49** showed potent activities against eight pathogens (MICs = 11.0−88.1 μM). | [Li et al., 2012] |
| stemphyperylenol (**50**) | *Botryosphaeria dothidea* | From the stem bark of *Melia azedarach* L., collected at Yangling, Shaanxi province, China. | Compounds **50** displayed potent antifungal activity against *A. solani* with MIC of 1.57 μM. | [Xiao et al., 2014] |
| preussomerins EG_1_ (**51**), EG_2_ (**52**), EG_3_ (**53**) | *Edenia gomezpompae* | From the leaves of *Callicarpa acuminata* from the ecological reserve El Eden, Mexico. | Compounds **51−53** displayed potent growth inhibition against four phytopathogens (IC_50_, 57.5−447.4 μM). | [Macías et al., 2008] |
| fonsecinone A (**54**) | *Aspergillus* sp. KJ-9 | From the stem bark of *Melia azedarach* collected at Yangling, Shaanxi province, China. | Compound **54** had marked inhibition of *M. grisea*, *B. cinerea*, and *A. solani*, with MICs from 6.25 to 25 μM. | [Xiao et al., 2014] |
| macrosporin (**55**) | *Phoma* sp. L28 | From the leaves of the mangrove plant *Myoporum bontioides*, collected from Leizhou peninsula, Guangdong Province, China. | Compound **55** showed broad-spectrum antifungal activity, with MIC values ranging from 13.2 to 252.1 μM. | [Huang et al., 2017] |
| (4*S**,5*S**)-2,4,5-trihydroxy-3-methoxy-4-methoxycarbonyl-5-methyl-2-cyclopenten-1-one (**56**), 4-chloro-1,5-dihydroxy-3-hydroxymethyl-6-methoxycarbonyl-xanthen-9-one (**57**) | *Alternaria* sp. R6 | From the root of a marine semi-mangrove plant *Myoporum bontioides*, collected from Leizhou peninsula, Guangdong Province, China. | Compounds **56** and **57** exhibited inhibitory activities against *F. graminearum* with MICs of 215.52 and 107.14 μM, while **57** inhibited C*. musae* with MIC of 214.29 μM. | [Wang et al., 2015] |
| cryptosporiopsin A (**58**), ponchonin D (**59**), hydroxypropan-2',3'-diol orsellinate (**60**), (−)-phyllostine (**61**) | *Cryptosporiopsis* sp. | From leaves and branches of *Zanthoxylum leprieurii* (Rutaceae), collected at Mont Kala near Yaoundé, Cameroon. | Compounds **58**−**61** exhibited motility inhibition of *P. viticola* zoospores, and also inhibited *P. ultimum*, *A. cochlioides*, and *R. solani*. | [Talontsi et al., 2012] |
| epicolactone (**62**), epicoccolide A (**63**), epicoccolide B (**64**) | *Epicoccum* sp. CAFTBO | From the stem bark and leaves of *Theobroma cacao* (Sterculiaceae) collected from Mount Kala, Centre Province, Cameroon. | Compounds **62**−**64** showed potent inhibitory effects on *P. ultimum*, *A. cochlioides*, and *R. solani*. | [Talontsi et al., 2013] |
| chaetomugilin A (**65**), chaetomugilin D (**66**) | *Chaetomium globosum* | From the seeds of *Panax notoginseng* that was collected at the Wenshan, Yunnan, China. | Compounds **65−66** showed moderate to potent inhibitory activities against *P. herbarum* and *E. nigrum*. | [Talontsi et al., 2013] |
| viburspiran (**67**) | *Cryptosporiopsis* sp. | From the shrub *Viburnum tinus* collected at Gomera. | Compound **67** was active against *Microbotryum violaceum* and *B. cinerea* with inhibition radius of 6 and 10 mm. | [Saleem et al., 2011] |
| chaetoglobosin X (**68**) | *Chaetomium globosum* L18 | From the leaves of *Curcuma wenyujin*, collected in Zhejiang Province, Wenzhou, China | Compound **68** exhibited potent activity against *E. turcicum*, *F. oxysporium*, and *C. lunata* with an MIC of 7.5 μM and showed moderate activity against *F. graminearum* and *F. moniliforme* with an MIC of 15.1 μM. | [Wang et al., 2012] |
| 2-methyl-5-methoxy-benzopyran-4-one (**69**), (2'*S*)-2-(propan-2'-ol)-5-hydroxy-benzopyran-4-one (**70**) | *Curvularia* sp. | From the leaves of *Ocotea corymbosa* collected in Araraquara, São Paulo, Brazil. | Compounds **69** and **70** exhibited moderate antifungal activity against *C. sphaerospermum* and *C. cladosporioides* with a detection limit of 10 μg. | [Teles et al., 2005] |
| phomochromone A (**71**), phomochromone B (**72**), phomotenone (**73**) | *Phomopsis* sp. | From the plant *Cistus monspeliensis*. | Compounds **71**−**73** showed moderate antifungal activity against *Microbotryum violaceum*. | [Ahmed et al., 2011] |
| 5-carboxy-6-hydroxy-3-methyl-3,4-dihydroisocoumarin (**74**) | *Xylaria* sp. | From *Casearia sylvestris* leaves collected in São Paulo State, Brazil. | Compound **74** exhibited potent antifungal activities against *C. cladosporioides* and *C. sphaerospermum* at 10 μg. | [Chapla et al., 2018] |
| (3*S*)-3,6,7-trihydroxy-α-tetralone (**75**) | *Phoma* sp. ZJWCF006 | From tubers of *Arisaema erubescens* collected from Wencheng County of Zhejiang Province, China. | Compound **75** showed growth inhibition against *F. oxysporium* and *R. solani* with EC_50_ values of 2.1 and 0.3 mM. | [Wang et al., 2012] |
| diaporthelactone (**76**), 7-hydroxy-4,6-dimethy-3*H*-isobenzofuran-1-one (**77**), 7-methoxy-4,6-dimethyl-3*H*-isobenzofuran-1-one (**78**) | *Phomopsis* sp. A123 | From the foliage of the plant *Kandelia candel* collected from the mangrove nature conservation area of Fugong, Fujian, China. | Compounds **76** and **77** displayed antifungal activity against *A. niger* with MICs of 243 μM and 485 μM, while **78** inhibited the growth of *A. alternaria* with an MIC of 500 μM. | [Zhang et al., 2014] |
| 5-methoxy-7-hydroxyphthalide (**79**), (3*R*,4*R*)-*cis*-4-hydroxymellein (**80**) | unidentified Ascomycete | From the plant *Meliotus dentatus* from the coastal area of the Baltic Sea, Ahrenshoop, Germany. | Compounds **79** and **80** showed antifungal activity against *Microbotryum violaceum* with the radius of zone of inhibition of 7 and 8 mm. | [Hussain et al., 2009] |
| 2,6-dihydroxy-2-methyl-7-(prop-1*E*-enyl)-1-benzofuran-3(2*H*)-one (**81**) | *Verticillium* sp. | From the roots of *Rehmannia glutinosa* collected from Wushe County, Henan Province, China. | Compound **81** inhibited biomass accumulation at 4.4 μM on *Septoria* sp. and *Fusarium* sp. | [You et al., 2009] |
| pestafolide A (**82**), pestaphthalide A (**83**), pestaphthalide B (**84**) | *Pestalotiopsis foedan* | From the branches of an unidentified tree near Dongzai, Hainan Province, China. | Compounds **82**−**84** showed moderate antifungal activity against *C. albicans*, *G. candidum*, and *A. fumigatus*. | [Ding et al., 2008] |
| griseofulvin (**85**) | *Nigrospora* sp. LLGLM003 | From a root of *Moringa oleifera* collected in Xiamen municipality, Fujian Province, China. | Compound **85** exhibited potent activity against *B. cinerea* and *C. orbiculare* with the EC_50_ of 0.6 and 1.4 μM. | [Zhao et al., 2012] |
| pestalotheol E (**86**), F (**87**), G (**88**), H (**89**) | unidentified Ascomycete | From the tree *Arbutus unedo*. | Compounds **86**−**89** showed moderate activity against *Microbotryum violaceum*. | [Qin et al., 2011] |
| pyrenocine K (**90**), pyrenocine M (**91**) | *Phomopsis* sp. | From the plant *Cistus salvifolius*. | Compounds **90** and **91** showed antifungal activity against *M. violaceum* with inhibition zone of 5 mm. | [Hussain et al., 2012] |
| koningiopisin C (**92**) | *Trichoderma koningiopsis* | From a healthy plant of *Panax notoginseng* collected from Wenshan, Yunnan Province, China. | **92** exhibited antifungal activity against *Plectosphaerella cucumerina* with an MIC of 57.1 μM. | [Liu et al., 2016] |
| (5-hydroxy-1-(3-oxo-but-1-ynyl)-7-oxa-bicyclo[4.1.0]hept-3-en-2-one) (**93**), monocerin (**94**) | *Drechslera* sp. strain 678 | From the roots of an Australian native grass *Neurachne alopecuroidea*. | Compounds **93** and **94** were active against *B. cinerea* and *S. sclerotiorum*. | [d’Errico et al., 2020] |
| epoxydine B (**95**), epoxydon (**96**), (4*R*,5*R*,6*S*)-6-acetoxy-4,5-dihydroxy-2-(hydroxymethyl)cyclohex-2-en-1-one (**97**) | *Phoma* sp. | From the halotolerant plant *Salsola oppostifolia*. | Compounds **95**−**97** were antifungal against *M. violaceum*. | [Qin et al., 2010] |
| ficipyrone A (**98**) | *Pestalotiopsis fici* | From the branches of *Camellia sinensis* in Hangzhou, China. | **98** displayed antifungal activity against *G. Zea*, with an IC_50_ value of 15.9 μM. | [Liu et al., 2013] |
| phomopsinones A−D (**99**−**102**) | *Phomopsis* sp. | From the stems of *Santolina chamaecyparissus* from Sardinia. | **99** showed potent antifungal activity against *B. cinerea*, *Pyricularia oryzae*, and *Septoria tritici*, while **102** was active against *B. cinerea* and *S. tritici*. | [Hussain et al., 2012] |
| nigrosporalactone (**103**), phomalactone (**104**) | *Nigrospora* sp. YB-141 | From stems of *Azadirachta indica* collected in Yuanjiang County, Yunnan Province, China. | Compounds **103** and **104** were active against *B. cinerea*. | [Wu et al., 2009] |
| 5-hydroxyramulosin (**105**) | Unidentified | From the plant *Cinnamomum mollissimum* which was sampled at Universiti Kebangsaan Malaysia, Selangor, Malaysia. | **105** inhibited the fungal pathogen *A. niger* (IC_50_ 7.9 μM). | [Santiago et al., 2012] |
| phomopoxides B (**106**), C (**107**), D (**108**), F (**109**), G (**110**) | *Phomopsis* sp. YE3250 | From the fresh stems of *Paeonia delavayi* in Songming County, Yunnan, China. | Compounds **106**−**110** were active against five pathogenic fungi with MICs of 46.5−372.1 μM. | [Huang et al., 2018] |
| fusidilactone D (**111**), fusidilactone E (**112**) | *Fusidium* sp. | From the leaves of *Mentha arvensis*. | Both compounds had moderate activity against *M. violaceum*. | [Qin et al., 2009] |
| sporothriolide (**113**) | *Nodulisporium* sp. A21 | From leaves of *Ginkgo biloba* collected in Nanjing, Jiangsu Province, China. | **113** was potent antifungal against *R. solani* and *S. sclerotiorum*. | [Cao et al., 2016] |
| (2*Z*,4*E*)-6(acetyloxy)-5-formyl-7-oxoocta-2,4-dienoate (**114**) | *Lophodermium* sp. | From the needles of superior *Pinus strobus* (eastern white pine) trees originating in New Brunswick, Canada. | **114** was antifungal against *M. violaceum* with MIC of 2 μM. | [Sumarah et al., 2011] |
| terrein (**115**) | *Aspergillus terreus* JAS-2 | From medicinal plant *Achyranthus aspera*. | **115** inhibitied *Bipolaris Sorokiniana* (57.14%), *A. flavus* (52.5%), and *A. alternate*. | [Goutam et al., 2017] |
| bicolorins A (**116**), B (**117**), D (**118**), E (**119**), G (**120**), H (**121**) | *Saccharicola bicolor* | From the fresh root of *Bergenia purpurascens* collected from the Duilong Country of the Tibet Autonomous Region, China. | Compounds **116**−**121** possessed weak to moderate activity against five pathogenic fungi with MICs of 26.8−380.9 μM. | [Zhao et al., 2020] |
| rhizopycnin D (**122**), TMC-264 (**123**) | *Rhizopycnis vagum* Nitaf22 | From a three-year-old tobacco *Nicotiana tabacum* grown in the greenhouse of the campus at China Agricultural University. | **122** and **123** strongly inhibited the spore germination of *M. oryzae* with IC_50_ values of 33.9 and 34.1 μM. | [Lai et al., 2016] |
| 3-(5-oxo-2,5-dihydrofuran-3-yl) propanoic acid (**124**) | *Aspergillus tubingensis* | From the stem of *Decaisnea insignis* collected from Qinling Mountain, Shaanxi, China. | **124** exhibited potent antifungal activity against *F. graminearum* with MIC value of 102.6 μM. | [Yang et al., 2019] |
| piliformic acid (**125**) | *Xylaria* sp. | From leaves of guarana plant with anthracnose symptoms (necrotic lesions), collected at Manaus-AM (03°22′5) and Maués-AM (57°42′0). | **125** had antifungal activity against *C. gloeosporioides* with an MIC of 2.92 μM. | [Elias et al., 2018] |
| ferulic acid (**126**) | *Aspergillus* sp. | From leaves of *Moringa oleifera* growing in Anambra State, South-Eastern Nigeria. | **126** showed weak antifungal activity against *A. niger* with an inhibition zone diameter of 2 mm | [Abonyi et al., 2018] |
| cordycepsidone A (**127**) | *Cordyceps dipterigena* | From a healthy leaf of *Desmotes incomparabilis* collected in Coiba National Park, Veraguas, Panama. | **127** showed potent activity against *Giberella fujikuroi* (MIC, 23.3 μM) and *Pythium ultimum* (MIC, 3.4 μM). | [Varughese et al., 2012] |
| botryorhodine A (**128**), B (**129**) | *Botryosphaeria rhodina* | From the herb *Bidens pilosa* collected at the museum of agriculture in Cairo, Egypt. | **128** and **129** were active against *A. terreus* with MICs of 26.03 and 49.70 μM. | [Abdou et al., 2010] |
| colletotric acid (**130**) | *Colletotrichum gloeosporioides* | From the stem of *Artemisia mongolica* collected in Zijin Mountain in Nanjing, China. | **130** was inhibitory to the fungus *Helminthsporium sativum* with an MIC of 95.4 μM. | [Zou et al., 2000] |
| penicillither (**131**), methyl dichloroasterrate (**132**) | *Aspergillus capensis* CanS‑34A | From *Brassica napus*, collected in Wuhan city, Hubei Province of central China. | Both compounds inhibited four fungi (*B. cinerea*, *M. fructicola*, *S. sclerotiorum*, and *S. trifoliorum*) with the EC_50_s of 21.7−151.2 μM. | [Qin et al., 2019] |
